# Obesity Among Young Adults in Developing Countries: A Systematic Overview

**DOI:** 10.1007/s13679-016-0187-x

**Published:** 2016-02-16

**Authors:** Amudha Poobalan, Lorna Aucott

**Affiliations:** Public Health Nutrition Group, University of Aberdeen, Polwarth Building, Foresterhill, Aberdeen, AB25 2ZD UK; Medical Statistics Group, Polwarth Building, Foresterhill, Aberdeen, AB25 2ZD UK

**Keywords:** Obesity, Young adults, Developing countries, Systematic

## Abstract

This article discusses the overweight/obesity situation among young adults in developing countries. For this target population, obesity prevalence ranges from 2.3 to 12 %, and overweight is 28.8 %, mostly affecting females. Weight is now increasing during this life stage of transition at a higher rate, 1 kg/year, than in developed countries. Maternal factors and early childhood socioeconomic status are associated with BMI in young adults along with changing environmental and behavioural factors in some low and middle income countries, brought about by demographic and socioeconomic transitions. Young adults with ‘normal weight’ obesity need identification using other convenient low cost measures (skin folds or waist circumference) along with BMI. Obesity prevention or management interventions were not identified, but clearly needed to help stem the obesity pandemic. Young people generally give little priority to their future health, so such interventions need to be conducted at some optimal age, be innovative, country specific and culturally acceptable.

## Introduction

Young adults between the ages of 18–25 are in a period of ‘transition’ from adolescence to adulthood. Until recently, it was perceived that obesity mostly affected middle age adults. However, a steadily increasing trend of obesity among young adults, especially college and university students, is becoming evident [1, 2]. Many young adults undergo significant lifestyle changes such as leaving home, going to university/college [3], starting work, developing relationships, possibly cohabiting or marrying [4], potentially experiencing pregnancy [5] and child rearing [6]. These transitions are seen as a time of displacement, when young people feel a sense of ‘loss’ and ‘discontinuity of their identity’ as they leave behind familiar contexts [7] and take on new ventures. These key time points in the life course, make young adults vulnerable to energy imbalance often leading to weight gain, which may not be concerning at the time but later accumulates. It is known that the interaction of social, psychological and biological factors that happen during these transition years may make them vulnerable to many risk taking behaviours [8••], but can be countered towards positive behavioural changes given constructive childhood and adolescent experiences [9, 10]. Nonetheless both positive and negative health behaviours established during this transition to adulthood often persist later into life [11] and hence this is a critical stage in a person’s life course [12]. It is also a time when inequalities of opportunity can be stark, with the transition period being particularly risky for those who are already vulnerable [13•, 14]. In spite of this recognition, young adults have been neglected both in terms of research and policy until recently [15].

Developed countries are only now beginning to recognise 18–25 year olds as a ‘vulnerable group’ for unhealthy lifestyles leading to overweight and obesity [16, 17•]. In contrast, among developing countries, obesity among all age groups was not even considered a public health problem until the early 1980–1990s and was perceived as a problem of developed countries. However, in the last two decades, several nutritional and socioeconomic transitions have changed the anthropometric measurements and health patterns of populations within developing countries [18••]. While conditions such as TB, diarrhoea and undernutrition are far from resolved, particularly in a few developing countries, the toll of obesity along with other NCD’s is escalating and seen as challenging to the health care infrastructure and health care delivery system of developing countries [19]. This current obesity epidemic is more pronounced in developing countries undergoing rapid epidemiological transitions (demographic, social and economic) with the anticipated tripling of obesity incidence in the near future [20••, 21]. This review aims to discuss the state and implications of overweight and obese young adults, who are in transition from adolescence to adulthood, specifically in the context of developing countries.

## Definition of Young Adult and Methodology

A systematic search was conducted in four electronic bibliographic databases (*Medline*, *Embase*, *PsychINFO and Scopus*) using a robust search strategy. The review included published literature from the start of the databases until May 2015, with no language restrictions. Appropriate MeSH terms and text words were used in the search strategy to capture all published literature on overweight and obesity among young adults in the developing (low and middle income) countries. Unfortunately, the term ‘young adults’ is often used to describe studies conducted among those ranging anywhere from 15 up to 50 years old. The definition of young adults is still ambiguous and intangible, with various definitions based on the stages of human development and sociological perspectives, defined by maturational, occupational, sexual and emotional indices [22–24]. However, for this review in the context of obesity, a tighter age range was required and so studies were only included if it was clearly stated in the title or abstract that participants were within the age range of 16–30 years; it was felt this would capture the age of transition from adolescence to adulthood for most global social settings. Studies were excluded if the mean age was over 30 years but also those with the wider age range (15–49 years old), which when inspected previously tended to have mean ages older than the group of interest [17•].

## Prevalence of Overweight and Obesity and consequences

Prevalence of obesity among young adults in developing countries range from 2.3 to 12 %, with overweight as high as 28.8 % (Table 1). This is already more or less in line with developed countries where current estimates of the prevalence of being overweight or obese (OW/obesity) ranges from 22 to 35 % among the 18–23 years old in UK and the USA [2, 25]. Studies among Indian, Egyptian and Ugandan females showed significantly higher levels of overweight and central/truncal obesity [26–29]. In cross sectional studies, for both males and females, BMI, waist circumference and waist hip ratio increase with age from 15–19 year olds to 20–29 year olds, but this was observed especially among males [30]. Increasing trends with age were observed not only in weight gain but also for other cardiovascular risk factors [30, 31] (Table 2). In one study where weight was observed over two different 5 year periods (from 20–29 year olds to 25 to 34 years old), 42 % of men and 56 % of women gained more than 5 kg [31]. They found this corresponded to a mean increase in % body fat of +7.6 % points in men and +5.9 % points in women and waist circumference of +10.2 cm in men and +7.7 cm in women. The % body fat changes were also associated with changes in blood pressure and the total:HDL cholesterol ratio, and resulted in an increased odds of developing metabolic syndrome: an OR 1.19 (95 % CI 1.08–1.31) for a 1.31 unit change in % body fat and an OR 1.13 (95 % CI 1.07–1.20) for a 1.20/cm change in waist circumference. Recent observations suggest that 23 % of Saudi Arabian and 16.7 % of Egyptian 18–25 year old young adults were already at an increased risk of developing fatal cardiovascular disease within 10 years [32]. Overweight and obesity also has an impact on respiratory functions within this group with decrease in slow and forced vital capacity [33].

**Table 1:** Prevalence of Overweight and Obesity among young adults

Author, Country, Year	Age	Setting	Sample size	Prevalence			
Gupta R et al. India 2009 [30]	15–19 years 20–29 years	Schools and Colleges (Delhi and Jaipur)	Males 15–19 years = 699 20–29 years = 175 Females 15–19 years = 591 20–29 years = 311	Variables (at risk values)	Males	Females	
				BMI (≥25 kg/m^2^)			
				15–19 years	53 (7.6 %)	52 (8.8 %)	
				20–29 years	20 (11.5 %)	23 (7.4 %)	
				BMI (≥30 kg/m^2^)			
				15–19 years	7 (1 %)	5 (0.8 %)	
				20–29 years	7 (4 %)	7 (2.3 %)	
				Waist circumference (>90 cm/80 cm)			
				15–19 years	32 (4.6 %)	36 (6.1 %)	
				20–29 years	20 (11.4 %)	31 (10.0 %)	
				Wait hip ratio (>0.9/0.8)			
				15–19 years	34 (4.9 %)	85 (14.4 %)	
				20–29 years	33 (18.9 %)	107 (34.4 %)	
Baalwa J et al. Uganda 2010 [26]	18–30 years Mean age 19.0 ± 1.4 years	Secondary schools in one urban and one rural area	683 Young adults Males = 338 Females = 345	Variables (at risk values)	Males	Females	
				Overweight (≥25 kg/m^2^)	3.3 %	17.4 %	
				Obese (≥30 kg/m^2^)	1.8 %	2.9 %	
Olusanya JO Nigeria 2011 [28]	16–27 years	Tai Solarin University	371 Students males = 151 Females = 220	Variables (at risk values)	Males	Females	
				Overweight and obesity (≥25 kg/m^2^)	5.1 %	10 %	
Dhruv S et al. India 2012 [62]	18–26 years Mean age 21	University of Baroda	1303 Females	Variables (at risk values)	Asia pacific cut off	WHO cut off	
				Overweight	9 %	10.1 %	
				Obese	12.6 %	2.5 %	
				Waist circumference (≥80 cm)		12.7 %	
				Waist Hip Ratio (WHR) ≥0.8 cut off		21.2 %	
Saleem et al. Pakistan 2013 [55]	Mean age 19.59 ± 1.7 years	University undergraduate students	Males = 856 Females = 544	Variables (at risk values)	Males	Females	
				Overweight (≥23–27.4 kg/m^2^)	12.7 %	8.3 %	
				Obese (≥27.4 kg/m^2^)	12.9 %	5.7 %	
Peltzer K et al. 22 countries 2014 [34]	16–30 years Mean age 20.8, SD 2.6	University undergraduate students from 22 countries	15,746 Students	Variables (at risk values)	Males	Females	
				Overweight ( ≥23–27.4 kg/m^2^)	18.9 %	15.2 %	
				Obese (≥27.4 kg/m^2^)	5.8 %	5.2 %	
Pengpid et al. India 2014 [29]	17–20 years Mean age 18.2 years (SD 1)	University students	Total 800 Males = 541 Females = 259	Variables (at risk values)	Males	Females	
				Overweight ( ≥23–27.4 kg/m^2^)	28.8 %	22.4 %	
				Obese (≥27.4 kg/m^2^)	12 %	12 %	
				Waist circumference (>90 cm in men/80 cm in women)	12 %	25.5 %	
Faarag M et al. Egypt 2014 [27]	Mean age 20.2 ± 1.8 years	10 Universities	Total 895	Variables (at risk values)	Males	Females	Total
				Obese (≥30Kg/m^2^)	NR	NR	13 %
				Truncal obesity (Waist to height ratio)	38.8 %	51.7 %	
Torun B et al. Guatemala 2002 [37]	19–29 years old	Community	Males = 237 Females = 236	Variables (at risk values)	Non-migrants	Migrants	*P* values
				BMI (≥25Kg/m^2^)			
				Females	53 (30 %)	12 (28 %)	NS
				Males	7 (7 %)	12 (16 %)	0.05
				BMI (≥30Kg/m^2^)			
				Females	17(10 %)	3(7 %)	NS
				Males	3(3 %)	0(0 %)	NS
				Wait hip ratio (M ≥ 0.90; F ≥ 0.85)			
				Females	37 (22 %)	1 (2 %)	0.05
				Males	24(25 %)	12(16 %)	NS
Bhongir AV India 2011 [36]	18–22 years	University (medical students in Hyderabad)	Urban = 116 Rural = 116	Variables (at risk values)	Rural	Urban	P values
				BMI (≥25Kg/m^2^)- overweight and obese			
				Total	4 (3.5 %)	37(31.9 %)	<0.001
				Females	2 (3.5 %)	22 (37.9 %)	<0.001
				Males	2 (3.5 %)	15 (25.9 %)	<0.001
				Wait hip ratio (M ≥ 0.90; F ≥ 0.83)			
				Total	7 (6.0 %)	17 (15.5 %)	NS
				Females	6 (10.3 %)	6 (10.3 %)	NS
				Males	1 (1.7 %)	11 (19.0 %)	<0.001

*NR* Not Reported, *NS* Not Significant, *BMI* Body Mass Index

**Table 2:** Prevalence of Cardiovascular Risk factors among young adults

Author, Country, Year	Age	Setting	Sample size	Prevalence			
Gupta R et al. India 2009 [30]	15–19 years 20–29 years	Schools and Colleges (Delhi and Jaipur)	Males 15–19 years = 699 20–29 years = 175 Females 15–19 years = 591 20–29 years = 311	Variables (at risk values)	Males	Females	
				Diabetes mellitus (≥126 mg/dl)			
				15–19 years	–	–	
				20–29 years	1 (0.6 %)	–	
				Hypertension (value cut-offs not reported)			
				15–19 years	16 (2.3 %)	2 (0.3 %)	
				20–29 years	10 (5.7 %)	5 (1.6 %)	
				HDL cholesterol (<40 mg/dl)			
				15–19 years	56 (8.0 %)	268 (45.3 %)	
				20–29 years	30 (17.1 %)	120 (38.6 %)	
				LDL cholesterol (≥130 mg/dl)			
				15–19 years	17 (2.4 %)	19 (3.2 %)	
				20–29 years	24 (13.7 %)	18 (5.8 %)	
				Total:HDL cholesterol ratio (≥4.5)			
				15–19 years	26 (3.7 %)	28 (4.7 %)	
				20–29 years	30 (17.1 %)	22 (7.1 %)	
Dhruv S et al. India 2012 [62]	18–26 years Mean age 21	University of Baroda	1303 females	Variables (at risk values)		Females	
				Metabolic syndrome—IDF definition		2.4 % (4.1 % by WHO definition)	
				Low HDL cholesterol (<40 mg/dl)		40.3 %	
				High LDL cholesterol		12.1 %	
Madeira FB et al. Brazil 2013 [39]	23–25-year-olds	Young adults from 1978/79 Ribeirao Preto birth cohort	1222 Normal weight obesity (NWO) young adults Males = 546 Females = 676	Variables (at risk values)	Males	Females	*P* value
				Metabolic syndrome—JIS definition	3.1 %	0.9 %	0.004
				Waist circumference (>90 cm in men/80 cm in women)	6.8 %	6.5 %	0.852
				Low HDL cholesterol (<40 mg/dl for men and <50 mg/dl in women)	31.2 %	38 %	0.014
				High blood pressure (≥130/85 mmHg)	28.9 %	3.3 %	<0.001
				Fasting glucose (≥100 mg/dl)	4.4 %	1.7 %	0.004
				HOMA2 insulin resistance (>1.8)	2.0 %	2.6 %	0.539
Bhongir AV India 2011 [36]	18-22 yrs	University (medical students in Hyderabad)	Urban = 116 Rural = 116	Variables (at risk values)	Rural	Urban	*P* values
				Fasting glucose (≥100 mg/dl)			
				Total	2 (1.7 %)	0 (0 %)	NS
				Females	1 (1.7 %)	0 (0 %)	NS
				Males	1 (1.7 %)	0 (0 %)	NS
				Hypertension (≥130/80 mmHg)			
				Total	15 (12.9 %)	18 (15.5 %)	NS
				Females	6 (10.3 %)	2 (3.5 %)	NS
				Males	9(15.8 %)	16 (27.6 %)	NS
				Total cholesterol (≥200 mg/dl)			
				Total	1 (0.9 %)	7 (6 %)	<0.05
				Females	1 (1.7 %)	4 (6.9 %)	NS
				Males	0 (0 %)	3 (5.2 %)	NS
				Low HDL (<50 mg/dl)			
				Total	69 (59.5 %)	66 (56.9 %)	NS
				Females	49 (85.0 %)	46 (79.3 %)	NS
				Males	21 (35.6 %)	20 (34.5 %)	NS
				Physical exercise (>4 hrs a week)			
				Total	58 (50.0 %)	42 (36.2 %)	NS
				Females	33 (56.9 %)	16 (27.6 %)	<0.05
				Males	25 (43.1 %)	26 (44.8 %)	NS
Torun B et al. Guatemala 2002 [37]	19–29 years old	Community	Males = 237 Females = 236	Variables (at risk values)	Non-migrants	Migrants	*P* values
				Fasting glucose (>6.1 mmol/l)			
				Females	4 (2 %)	0 (0 %)	NS
				Males	3 (3 %)	1 (1 %)	NS
				Total cholesterol (>5.2 mmol/l)			
				Females	29 (17 %)	5 (12 %)	NS
				Males	5 (5 %)	8 (11 %)	NS
				Low HDL (<0.9 mmol/l)			
				Females	55 (33 %)	7 (17 %)	<0.05
				Males	40 (44 %)	36 (52 %)	NS
				Moderate physical exercise (MET/24 hr)			
				Females	32 (18 %)	6 (14 %)	NS
				Males	75 (73 %)	16 (21 %)	<0.05
				% Body fat (>23.9 in men; >29.9 in women)			
				Females	75 (43 %)	20 (47 %)	NS
				Males	4 (4 %)	3 (4 %)	NS

*HDL* high density lipoprotein, *LDL* Low Density Lipoprotein, *JIS* Joint Interim Statement of the *IDF* International Diabetes Federation, *HOMA* Homeostasis Model Assessment 2, *MET* Metabolic Equivalents

A study, looking across 22 low and middle income countries, was conducted among 15,746 undergraduate students from 22 universities with mean age of 20.8 years. This highlighted that overall 22 % of young adults were OW/obese, with men (24.7 %) more than women (19.3 %) [34]. They also found the average age of these OW/obese men was younger (16–19) compared to women (22 years or more). However, for obesity alone, in sub-Saharan Africa and in Latin America, females were more obese whereas in Asia and North Africa (except Egypt and Tunisia), male obesity was more than the female obesity. Whether this links to the social disadvantages that women might encounter in Asia/North Africa is not clear yet.

A concept of Normal Weight Obesity (NWO) has now emerged, where individuals have a normal BMI of between 18.5 and 24.9 kg/m^2^ but the sum of their triceps and sub-scapular skinfolds is >90th percentile which corresponds to >23.1 % body fat in men and >33.3 % body fat in women. This has now also been observed among young people in low and middle income countries; with a study of 1222 normal BMI young adults between the ages of 23 and 25 years in Brazil finding 111 had NWO [35••]. These individuals had an increased risk of the Metabolic Syndrome (OR 6.83, 95 % CI 2.84–16.83), increased waist circumference (OR 8.46, 95 % CI 5.09–14.04), low HDL (OR 1.65, 95 % CI 1.11–2.47), insulin resistance (OR 3.81, 1.57–9.28) and low Insulin sensitivity (OR 3.89, 95 % CI 2.39–6.33) (Table 2).

### Urban-Rural Variations

Economic progress and industrialisation in many developing countries has seen massive rural to urban transitions. This has led to many related changes in lifestyles, resulting in increased rates of obesity and cardiovascular risk factors. Among 18–22 year old Indian young adults, BMI was significantly higher in urban men and women than their rural counterparts with a trend of higher waist to hip (W:H) ratios amongt the urban young adults albeit significant only in males [36]. Total cholesterol was also significantly higher for urban young adults, and urban women were also significantly less physically active [36]. Also highlighted was that while 70 % of rural young and 13.8 % of urban young were underweight (data not shown), 3.5 % of rural youth and 31.9 % of urban young were overweight or obese (OW/obese). The tenfold increase in the prevalence of OW/obesity in the urban young is of concern. In contrast, in Guatemala [37], both urban women who migrated from rural areas (migrants) and rural (non-migrants) women tended to be overweight; however only urban men were more overweight than their rural counterparts. Irrespective of BMI, rural men and women had more abdominal fat than those in urban areas and all, regardless of gender or location had lower levels of physical activity. Rural (non-migrant) women significantly were seen to have lower HDL compared to migrants, which is another indicator of cardiovascular disease. In Uganda, no difference was found in the prevalence of overweight between urban and rural areas [26].

It could be speculated that in some countries, the differences between the rural and urban lifestyle has narrowed as globalisation blurs the boundaries of the socioeconomic divide. Among 21 year old Filipino young females, there was an interaction between urbanicity, a persons’ assets and being overweight [38•]. Using a measure of urbanicity based on assets, together with income and level of education [39], they found that for those at the lower end of the scale, a one point increase in assets was associated with a 29 % increase in being overweight (OR 1.29 95 % CI 1.01–1.65); however, at the highest level of urbanicity, the same increase in assets was only associated with a non-significant 8 % reduction in overweight (OR 0.92 95 % CI 0.76–1.11) [39].

## Influences and Risk Factors

Weight problems in young adults have been linked to factors in gestation and early development particularly in young adults in low to middle income countries. Associations between BMI and/or waist circumference of young adults have been found with maternal anthropometry, foetal nutritional insufficiency due to inadequate maternal nutrition, birth weight, birth order and early child growth [40–42]. A study by Adair [40] showed that a mother’s arm fat area during pregnancy is positively associated with higher birth weight (*β* = 0.421 in boys and 1.108 in girls) and higher BMIs when their offspring are 21 years old (*β* = 0.489 in young males and 0.783 in young females). Similarly, Dahly [41] showed that maternal height and arm fat area were positive predictors of central obesity for 21 year old Filipino males. Among Brazilians young adults (23 year olds), their mothers’ pre pregnancy BMI was positively correlated with their own BMI and waist: hip ratio (*p* < 0.0001) [42].

With respect to socioeconomic status (SES), higher parental SES at birth and improved childhood SES had significant association with increased BMI in males (*β* = 2.04) but was decreased for female young Filipino adults (*β* = −2.188) [40]. Socioeconomic status and college education at 21.5 years was positively associated with overweight and increased waist circumference of young adults within the Philippines [41]. Multivariate linear models showed that one Standard Deviation (SD) increase in SES was associated with 149 % increase in odds of having a higher waist circumference (OR 2.49; 95 % CI 1.65–3.76), although this was attenuated by low birth order [41]. First born children from the high socioeconomic groups in this study had low birth weight and but had a rapid post-natal gain. These children had the largest increase in childhood BMI which continued into young adulthood as shown by other studies [43, 44]. These studies also show that while inadequate maternal nutrition and poor birth outcomes such as low birth weight leading to stunted child growth might be common in developing countries, there are indicators that socioeconomic improvement in developing countries could lead to rapid post-natal weight gain. This then contributes to higher BMI in young adults as they grow up in developing world settings. Although there is evidence that maternal factors play a part in the offspring’s BMI in young adulthood, environmental and behavioural factors has to be considered.

The comparison by Peltzer and others [34] across 22 developing countries showed, that for both men and women, physically inactivity and childhood physical abuse increased the risk of OW/obese, more so in the *relatively* higher income countries. However this study highlighted some unexpected findings—those who consciously avoided fat and cholesterol were OW/obese. A potential explanation given by the authors was that those avoiding the fatty food were already self-reportedly obese. They also found, women with post-traumatic stress disorder and/or attending frequent religious activity tended to be more OW/obese. The former could potentially be linked to depression, a known obesity related factor [45–47], but the latter could be related with religious beliefs or cultural norms regarding body image among women. Some religious organisations will provide a social network for being physically active however this might vary. Similarly, religious doctrinal beliefs can dictate intake of specific foods, beneficial or detrimental. This variation in the impact of religious activities on diet and physical activity needs further exploration [48•]. As in developed countries leaving home can have an impact on weight status [3]; in India [29], young males living away from their parents were more likely to have central obesity (AOR 1.79, 95 % CI 1.04–3.07).

## Perceptions of Body Weight and Image

Perceptions of body weight and the influence of body image on self-esteem, happiness, wellbeing and health can consequently lead to obesity [49, 50]. In developed western cultures, thinness is associated with being attractive, elegant and being in control [51]. This perception is different and contrary in some developing countries [52] where being ‘big’ is a sign of prosperity and good health [53, 54]. In Pakistan [55], 43 % of young males and 41 % of females misjudged their weights. While underestimation was more common in males, overestimation was seen more in females. The highest rate of misjudgement were among overweight males compared to females (95.4 vs 80 % females), where males underestimated their weights eight times more compared to females (OR 8.054, 95 % CI 5.34–12.13). This weight misperception increased with age (1.114, 95 % CI 1.041–1.191). Among 21-year-olds in Nigeria, 40 % of overweight males perceived overweight as the ideal body image where as 100 % overweight females perceived normal weight as ideal body image [56•]. Similarly all the obese young men in this study perceived being overweight as ideal while 67 % of the obese females perceived normal weight as ideal. These views are more in line with western perceptions. This gender discrepancy of body image perception shows that the cultural boundaries are disappearing through globalisation and technological innovation and if unabated, might push the young men wanting to gain weight to attain the ‘ideal’ buff image, while women might desire to lose weight, which in western cultures has led to often unattainable goals.

## Discussion

Obesity is a growing global Public Health concern affecting people of all age groups and socioeconomic status. Lower and middle income countries (LMIC) are also experiencing a marked increase in overweight and obesity, and yet at the same time are continuing to face major economic difficulties and increased food prices. While disease associated with under-nutrition is still a major issue, these are now being overtaken by diseases linked to unhealthy lifestyles, overweight and obesity [18••]. In countries undergoing social and economic transitions, there is a shift to more affluent social structures with a need to identify themselves with modern society. However, cultural values in favour of large body size traditionally associated with fertility and prosperity still persists in some countries [57].

Young people living in countries making the socioeconomic shift, are particularly affected by the social and environmental factors such as financial independence, the easy availability of ‘ready to eat’ food stuffs, the growing numbers of fast food chain shops, which are all part of adopting western life culture. The now readily available readymade foods and high sweetened beverages are more convenient and highly desirable, especially to young people. These are high in calories and fat content, making young people susceptible to an obesogenic environment [58–60] and incline towards unhealthy habits [61]. Huang et al. showed that less than a quarter of USA college students (18–24 years old) consumed at least five servings of fruit and vegetable per day [59]. The increased consumption of fast foods, snacking and skipping breakfast in young adults all escalate the risk of being overweight or obese later on in life [28, 62, 63]. Further changes from active to sedentary lifestyles both in and outside the workplace, the lack of time due to work commitments, the long hours and day-night duty rotations along with psychological changes related to the material and social environments all make it difficult for young people in this age group to be additionally involved in any kind of physical activity [64, 65]. While there is evidence that maternal/early life factors influence the increased levels of obesity, the poor response to later life challenges such as the ever increasing obesogenic environment adds to the concern for countries with low resources with the inevitable increase in non-communicable diseases [66, 67]. Given many young people between the ages of 18–25 will also be becoming parents, educating them on these aspects also becomes crucial.

Most developing countries are seeing higher overweight/obesity prevalence among females, with some exceptions. Obesity increases with age but among young adults in developing countries the average weight gain of 1 kg/year [31] is greater than that observed in developed countries (0.4–0.9 kg/year) [68, 69]. With the increased obesity levels seen in such a short period of time between 16–25 years of age, a logical argument would be to intervene earlier, however, young people in transition are very different to their younger selves with parental influences. Between the ages of 16–25, they have the independence to take risks with their lifestyle behaviours and future health is not their priority. The ideal age to intervene for this heterogeneous age group is an issue faced by researchers for other conditions such as cervical cancer screening [70, 71••] but generally there is a self-realisation that they should consider improving their lifestyle at around the age of 21 [9].

Perceptions of body image are continually changing among young adults in developing countries [72, 73]. Studies have shown that those with accurate perceptions of their own weight are more in control of their behaviours [74, 75]. Consequently, efforts to promote positive and yet realistic body image is equally important in addition to healthy eating and exercise behaviours. Such changes in body sizes are also mediated by social factors as explored by Sobal [76]. They showed that SES was positively associated with obesity in developing countries but negatively in developed countries. However, as economic status shifts, which in some cases has been rapid, the obesity/SES relationship is changing among young people, whereby low SES groups are more prone to obesity [41]. The SES and obesity gradient is inversely shifting in the low and the middle income countries and is becoming more like high income countries, particularly for women [77].

The evidence of NWO in low and middle income countries, particularly among young adults, is of concern especially with the potential increased risk of cardiovascular disease. In some low and middle income countries, obesity is assessed only by BMI. This isolated use of BMI runs the risk of missing those who, in spite of having a normal BMI, are at risk of metabolic disorders later on. Ideally other measures such as DEXA scans would more accurately measure the body composition but even incorporating other more convenient low cost measures like skin folds or waist circumference would be valuable in assessing excess body fat in young individuals with normal BMI, given the metabolic changes are observed early in the life course [78].

It is worth noting that, in spite of a robust search of studies conducted among young adults in developing countries, all the included studies were either cross sectional or secondary analysis from birth cohorts and health surveys, which can only highlight the prevalence of obesity and some of the contributing factors and consequences of being overweight or obese. Most studies have identified that the rapid socioeconomic transitions in developing countries are having a huge impact on obesity levels and are contributing to cardiovascular diseases. This is now a leading cause of death in all regions of the world, and that more than 80 % of the global disease burden is in low and middle income countries [79, 80]. In spite of this recognition, with many studies suggesting the need for interventions and improvement in public health policies, we found no evidence of interventions being conducted among young adults in developing countries.

There are some limitations that need to be acknowledged. Our search terms were ‘18–25 year olds’ and ‘young adults in transition’ and consequently some studies conducted among University students that did not mention our specific age range could have been missed in our search. Further, this article has focussed on over-nutrition among young adults, however, the problems of under-nutrition, eating disorders and the double burden of over and under-nutrition in transition countries [62, 81], while acknowledged, have not been explored to the same extent.

## Conclusion

Young adults (18–25 year olds) are prone to overweight and obesity during the transition from adolescence to adult in developing countries as much as in developed countries. Levels of overweight and obesity together with other cardiovascular risk factors increase with age even within this age span (18–25 years old). Using other convenient low cost measures to assess body fat in addition to BMI to measure obesity might be useful to identify Normal Weight Obesity in young adults, which is especially important to counter the risk of metabolic imbalances in later life. Understanding the epidemiology of obesity and investigating the optimal time to intervene is crucial. We found no recorded obesity prevention interventions being conducted in developing countries within this age group. Culturally acceptable interventions based on evidence need to be developed and evaluated. In turn these will inform future policy makers to help plan effective strategies to prevent obesity among young people.
